# Massive Retroperitoneal Hemorrhage as an Initial Presentation of a Rare and Aggressive Form of Multiple Myeloma

**DOI:** 10.1155/2016/8206826

**Published:** 2016-01-14

**Authors:** Aydah Alawadhi, Laszlo Leb

**Affiliations:** Department of Medicine, Saint Vincent Hospital, 123 Summer Street, Worcester, MA 01608, USA

## Abstract

Multiple myeloma, a plasma cell neoplasm, presents most commonly with anemia, hypercalcemia, renal failure, and bone pain. Only few cases of clinical aggressive presentation associated with bleeding were reported in the medical literature. The reported cases included gastrointestinal bleeding and cardiac tamponade. Spontaneous retroperitoneal haemorrhage as initial presentation has not been so far reported. We hereby report a case of a 64-year-old female who was found to have catastrophic hemorrhage in the retroperitoneal region that extended into intrathecal space causing cord compression. The case posed a significant diagnostic and management dilemma. This case emphasizes the need to think broadly and include multiple myeloma in the diagnosis of unexplained massive retroperitoneal bleeding.

## 1. Introduction

Multiple myeloma, a plasma cell neoplasm associated with production of monoclonal paraprotein, is second in prevalence amongst hematologic malignancies [1]. The diagnosis of multiple myeloma requires the presence of at least 10% of plasma cells in the bone marrow or the presence of extramedullary plasma cell tumor and at least one of the following clinical manifestation due to plasma cell proliferation: anemia, renal insufficiency, bone lesions, and increased plasma calcium level. More recently in addition to the presence of a monoclonal protein the involved: uninvolved serum free light chain ratio more than or equal to 100 proved to be of diagnostic significance [2]. The clinical presentation with major spontaneous bleeding is uncommon and seldom has been reported in patients with multiple myeloma. Previous retrospective studies demonstrated that bleeding tendency is more common with IgM and IgA paraprotein and the bleeding tendency in such cases has been attributed mainly to the higher serum viscosity due to the accumulation of such immunoglobulins. However, even in such cases, severe symptomatic bleeding as initial manifestation of multiple myeloma has not been reported [3]. We hereby report a first case of IgG lambda multiple myeloma presenting acutely with severe retroperitoneal hemorrhage refractory to all medical interventions representing a challenging diagnostic and management dilemma.

## 2. Case Report

**Figure 1:**
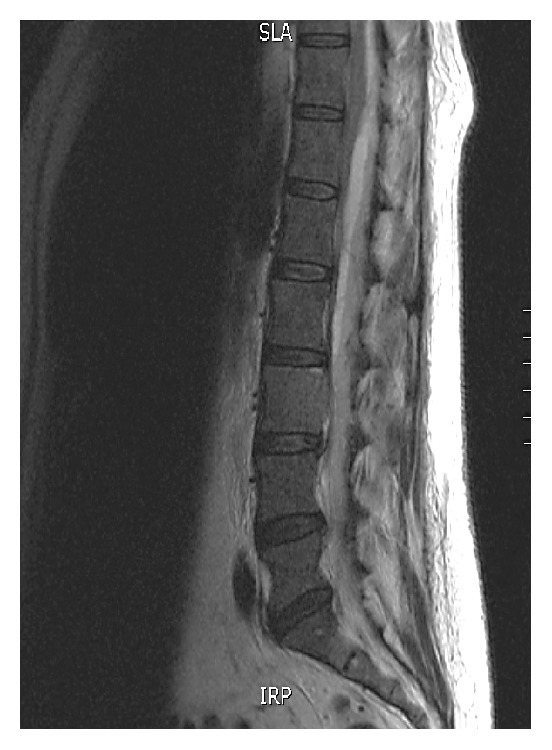
MRI of the lumbar spine in sagittal projection showing critical long segment spinal cord compression extending approximately from the T12, left paraspinal intramuscular hematoma level, inferiorly to the L5.

**Figure 2:**
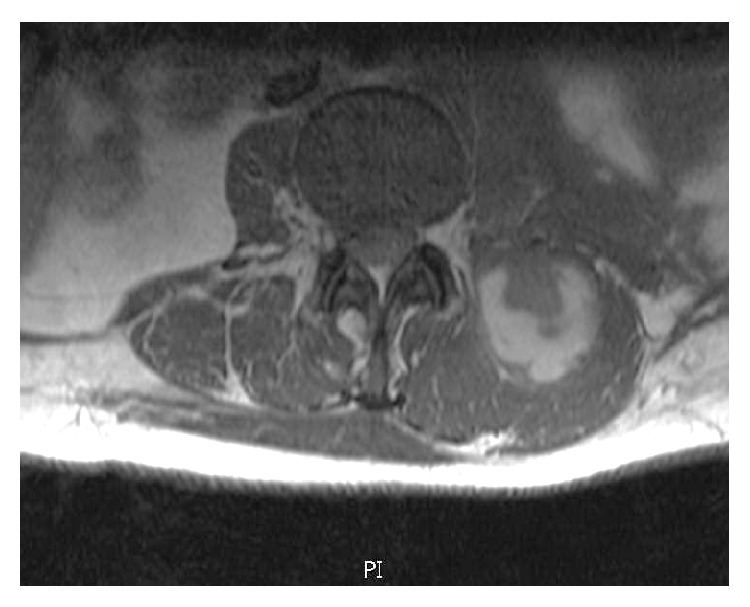
Axial section of MRI at the level of L5 showing left paraspinal intramuscular hematoma.

A 64-year-old female with past medical history of hyperlipidemia, osteoporosis, and diverticulosis presented to the emergency department with worsening, debilitating lower back pain of one-month duration. Further history revealed anorexia, low-grade fever, sweating, and shortness of breath for about 3 weeks and 10 pounds of weight loss. One month earlier, she visited the emergency department for right lower quadrant pain and was discharged with the diagnosis of diverticulitis. Laboratory test at that time did not reveal anemia, hypercalcemia, or decreased renal function. An abdominal computed tomography done at that time revealed incidental splenomegaly at 14.5 cm. Further evaluation was deferred to her primary care physician. At this presentation in the emergency department, the vital signs were stable, and she was pale and cachectic. Abdominal examination revealed rigidity, distention, tender splenomegaly, and Grey Turner's sign. Neurologic examination of lower extremities revealed weakness and numbness with hyperreflexia. The rest of the physical examination was unrevealing. Laboratory investigation showed the following: white blood cells of 13.8 × 1000/*μ*L, haemoglobin of 9 g/dL, and platelets of 101 × 1000/*μ*L. The peripheral smear showed rouleaux formation and plasma cells less than 10%. Earlier laboratory findings showed mild normocytic anemia, thrombocytopenia, and normal white blood count. Internal hemorrhage was greatly suspected given the clinical and laboratory findings. Other laboratory tests were significant for a creatinine of 2.5 mg/dL and a total calcium level of 9.0 mg/dL. Urinalysis was positive for blood and protein, 511 mg/dL. Prothrombin time was slightly elevated 14.2 sec and a normal activated partial thromboplastin time. Biochemical tests revealed a low total protein at 6.5 g/dL and serum albumin was low at 2.7 g/dL. Serum LDH was elevated at 240 U/L (60–160 U/L). Quantitative D-dimer was more than 0.5 mg/L (reference value is less than 0.5 mg/L). Fibrinogen was normal with a value of 194 mg/dL (193–423 mg/dL). Quantitative immunoglobulin showed an increased IgG at 2187 mg/dL and decreased IgA and IgM levels. Serum and urine immunofixation identified a monoclonal IgG lambda. CT abdomen revealed interval worsening of splenomegaly to 20 cm, multiple enlarged lymph nodes, and retroperitoneal haemorrhage extending from the left kidney to the left inguinal region. MRI of lumbar spine done emergently as the lower extremities showed progressive paralysis revealed critical long segment spinal cord compression extending from the T12 to L5 level secondary to medullary canal haemorrhage (Figures 1 and 2).

**Figure 3:**
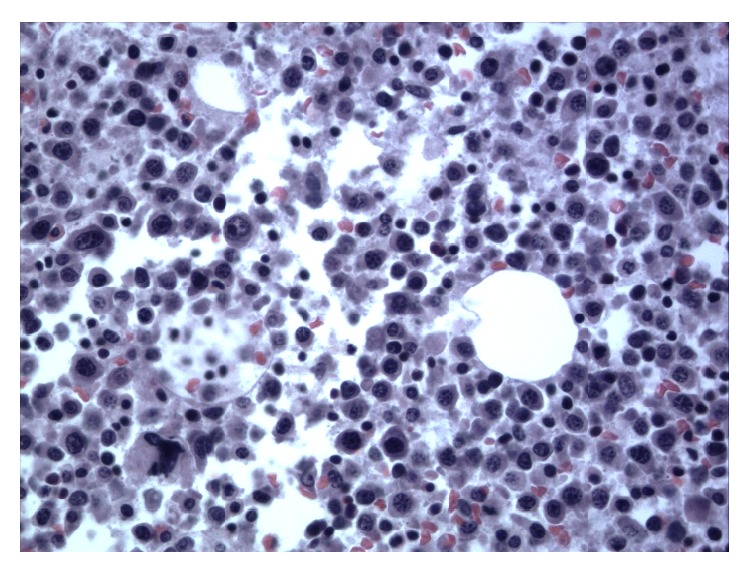
H&E staining of the bone marrow autopsy showing numerous plasma cell (estimated to be more than 10%).

**Figure 4:**
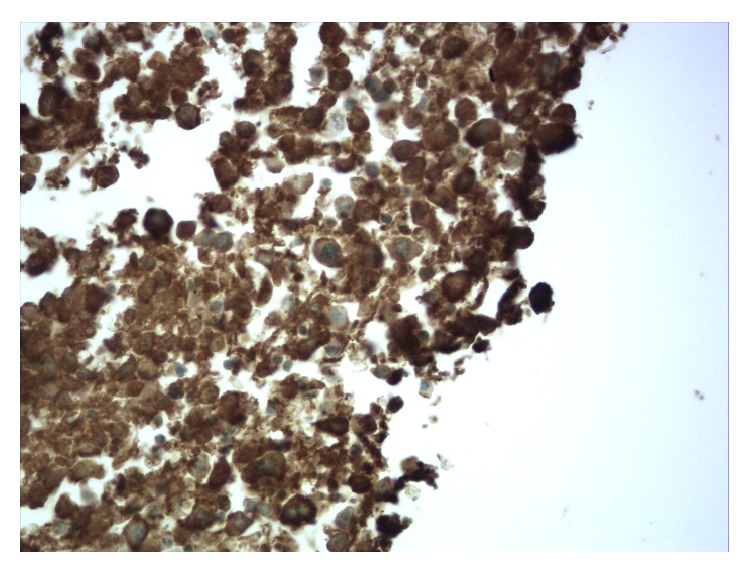
Postmortem bone marrow biopsy showing strongly positive Immunohistochemical stain for lambda immunoglobulin light chain within the plasma cell cytoplasm. (The IHC is suboptimal secondary to protein degradation during tissue autolysis in devitalized tissues.)

The patient was transferred to the ICU for developing severe haemorrhagic shock. Based on the clinical and laboratory data the diagnosis of multiple myeloma versus plasma cell leukaemia was considered. Plasma cell leukaemia was excluded as the peripheral blood showed less than 20% plasma cells which is the major criteria for diagnosing this condition. The cause of retroperitoneal bleeding in the absence of a bleeding disorder, vascular disease, and anticoagulant medications was considered to be related to the basic disease of multiple myeloma. Given the emergent and rapidly worsening condition, the patient was started on high dose corticosteroids and supportive measures including vasopressors and blood products including packed red blood cell, fresh frozen plasma, and platelets transfusion. The patient haematocrit and platelets count continued to drop precipitously. In 5 days, the hematocrit dropped from 9 g/dL to 5.6 g/dL and the platelets dropped from 101 × 1000/*μ*L to 56 × 1000/*μ*L despite supportive measure with blood products. Haematological consult was obtained. Due to the poor clinical status, bone marrow biopsy was deferred. Also, neurosurgery consultation was obtained to evaluate the developing cord compression. She was deemed to be a very poor surgical candidate. The ICU course was complicated with DIC confirmed by PT of 24 sec (9–12 sec), PTT of 47 sec (25–35 sec), and fibrinogen of 75 mg/dL (193–423 mg/dL), worsening acute kidney injury with creatinine increasing from 2.5 mg/dL to 5.5 mg/dL, and acute hypoxic respiratory failure requiring endotracheal intubation due to severe refractory anemia leading to high output cardiac failure, and the chest X-ray revealed bilateral pulmonary vascular congestion and prominent left-sided pleural effusion. Finally, seven days after admission, her code status was changed to do-not-resuscitate per her family and healthcare proxy, given her refractory illness and poor prognosis. In the same day, she developed cardiac arrest and expired. Autopsy was obtained and gross evaluation revealed large blood clot in the left pelvis and retroperitoneal space measuring 40 cm in greatest dimension and weighing 1060 g indicating that the most likely cause of death was a haemorrhagic shock probably due to underlying bleeding diathesis secondary to platelets dysfunction. Microscopic evaluation confirmed the diagnosis of multiple myeloma with more than 60% CD138 and lambda light chain positive plasma cell in the bone marrow (Figures 3 and 4). Also, microscopic evaluation of the spleen revealed heavy infiltration of plasma cells. Microscopic evaluation of the kidneys revealed light chain casts consistent with Bence Jones protein. Congo red staining was negative ruling out amyloidosis.

## 3. Discussion

This case illustrates a rare and aggressive form of multiple myeloma presenting with retroperitoneal hemorrhage leading to cord compression. This type of presentation of multiple myeloma is reported for the first time in the medical literature. One case of retroperitoneal haemorrhage developed, however, during a well-established multiple myeloma and was reported in 1991 and ultimately light chain amyloidosis was detected which might have contributed to the bleeding complication in that case [4]. In general, bleeding diathesis in multiple myeloma contributing to morbidity and death is often related to complications due to therapy, infection, renal failure, and invasive procedures rather than to the monoclonal paraprotein [5]. Plasma cell dyscrasia including multiple myeloma is associated with a bleeding diathesis in about 15% of the cases. Factors contributing to increased risk of bleeding are thrombocytopenia secondary to bone marrow failure, paraprotein induced platelets dysfunction, inhibition of fibrin monomers polymerization, monoclonal thrombin inhibitor, circulating heparin-like anticoagulant, and acquired Von Willebrand syndrome [6]. Due to the fulminant course of the disease, we were unable to perform in-depth studies of platelets function and coagulation tests. Such studies including thrombin time, coagulation factor assays, platelet aggregation studies, reptilase time, mixing studies using pooled normal plasma, and protamine should be valuable to establish the accurate cause of bleeding and therefore direct the appropriate therapy [5]. The presenting case brings to attention another cause of retroperitoneal bleeding and should urge in-depth workup of the cause of bleeding in patients with multiple myeloma eventually leading to a more appropriate management.
